# Treatment of Tracheal and Laryngeal Stenosis

**Published:** 1877-03

**Authors:** 


Treatment of Tracheal and Laryngeal Stenosis by Dilatation.—*By Thaou.*—Syphilis, small-pox, and typhoid fever, from their tracheal and laryngeal localizations, are the most frequent causes of contraction of the ærial conduit, and in these stenosis is most surely caused by submucous lesions.

Perichondritis is almost invariably observed, and in its train occurs necrosis of bone and anchylosis of the small articulation. It develops an exudation which leads to prominence of the mucous membrane, ulceration, or fibrous transformation, compressed and therefore inactive muscles, which undergo fatty degeneration. These processes are hardly evident to the laryngoscope. Sometimes the epiglottis is destroyed and falls upon the glottis, where, becoming adherent, it remains fixed in a vicious position.

It must be elevated with a curved sound in order to uncover the completely obstructed glottis. It is surprising that respiration should be possible through such a tortuous and occluded canal. Sometimes the tube is closed by a true diaphragm, more or less eccentrically perforated, and composed of a whitish cicatricial membrane.

The symptoms are referable to a distant date, when there was hoarseness, dyspnœa, and a sense of suffocation. Then tracheotomy does not cure; it saves life, but leaves behind the stricture which pre-existed, and a true fistule of a dangerous character. Existence at such a price is dear.

Before the tracheal tube can be removed, the natural passage way of the air must be restored. Liston (1827), Czermak (1851), Guerin, Richet, Busch, Brake, Gurlt, Burns, Dolbeau, Gerhardt, Treudelenberg, operated by instruments passed upward through the artificial opening.

The direct method (by the mouth) has been tried by only Roux, Despres, Thiersch and Navratil—always without success.

After two months' trial with the method of Schrotter, the author is disposed to recommend it highly. The former operates by the superior or natural passage, but the method of operating and the form of his instruments vary according to whether there (*a*) is or (*b*) is not a surgical opening in the neck.

(*a*) In this case the tracheal fistula is utilized only for the purpose of fixing in the larynx a dilating prism, introduced through the mouth. The prisms are zinc, triangular in shape, with rounded borders, and resemble in form the glottis. Inferiorily they present a small narrowed neck, surmounted by an equally small rounded head. The prism descends into the air passage and becomes engaged in the opened tracheal canula, toward its dome. A pair of slender forceps, capable of continuous pressure, is introduced through the neck, seizes the prism at its constricted part, and holds it in place. At its upper extremity the prism is provided with a stout thread. At the time of introduction this thread is made to pass through a long curved tube, when it is firmly fastened by a knot to the handle by which the tube is carried. Thus constituted, the apparatus represents an uninterrupted instrument, composed of a curved branch (the tube) and a terminal enlargement (the prism). By the aid of the laryngeal mirror, it is engaged in the larynx and fixed below by the tracheal forceps. The knot is then unfastened; the tube, which is no longer retained by the prism, is withdrawn, and the thread, which remains attached to the prism, is brought out of the mouth and attached to the ear. The patient or physician has but to draw upon the thread and open the forceps in order to extract the prism from the larynx.

Some education of the parts is requisite before proceeding to the dilatation, which is accomplished by the successive introduction of a series of prisms, numbered from one to twenty-four—the smallest being eight millimeters in one measurement and six in the other, the largest being twenty by sixteen. All are four centimeters long. The duration of the dilatation is from a few minutes at the outset to the entire night, the patient being finally able to sleep with the instrument *in situ*, because accustomed to its presence. The results are satisfactory to a surprising degree. Soon the knife or galvano-cautery may be used to divide a bridle, or various caustic applications may be made to localized swollen tissues. Sometimes the voice returns, or the patient can breathe and talk with the canula closed. Insuccess is due generally to obstructions lower down. Of course more recent cases afford the best results.

(*b*) In non-tracheotomized subjects the sounds are adapted to the different conditions, and the patients learn to use them themselves. The base of the tongue is depressed with the finger, and the instrument made to penetrate the larynx without hesitation. Schrotter employs also rubber sounds, somewhat longer than those described above, with a triangular laryngeal extremity and rounded borders. Lateral openings are made for the exit of mucous secretions. There is also an oval shield of hard rubber, so arranged that the expectorated secretions are directed to the floor, and not in the face of the operator. Often a previous incision of the obturator membrane by the knife or cautery is requisite before the introduction of the sound.—*Chicago Medical Journal and Examiner.*

